# EASIX for prediction of survival in lower-risk myelodysplastic syndromes

**DOI:** 10.1038/s41408-019-0247-z

**Published:** 2019-11-11

**Authors:** Almuth Merz, Ulrich Germing, Guido Kobbe, Jennifer Kaivers, Anna Jauch, Aleksandar Radujkovic, Manuela Hummel, Axel Benner, Maximilian Merz, Peter Dreger, Thomas Luft

**Affiliations:** 1 Department of Internal Medicine V, University Hospital Heidelberg, Heidelberg, Germany; 2 Department of Hematology and Oncology, University Hospital Dusseldorf, Dusseldorf, Germany; 3 Department of Human Genetics, University Hospital Heidelberg, Heidelberg, Germany; 4 Division of Biostatistics, German Cancer Research Center Heidelberg, Heidelberg, Germany

**Keywords:** Risk factors, Myelodysplastic syndrome

## Abstract

Patients with myelodysplastic syndromes (MDS) are at risk of early death from cardiovascular complications due to the link between clonal hematopoiesis and endothelial dysfunction. EASIX (Endothelial Activation and Stress Index) has been established to predict endothelial complications after allogeneic transplantation. We investigated the impact of EASIX measured at first diagnosis on survival of patients with lower- and higher-risk MDS (no allogeneic transplantation) in two independent institutions: *n* = 192 (training cohort) and *n* = 333 (validation cohort). Serum markers of endothelial cell distress were measured and correlated to EASIX. While no effects of EASIX on survival were observed in higher-risk patients, EASIX was associated with shorter survival in patients with lower-risk MDS in both cohorts (univariate: Cohort I: hazard ratio (HR): 1.46; 95% confidence interval (CI) 1.24–1.71; *p*-value < 0.001/Cohort II: HR 1.31 [1.17–1.48]; *p*-value < 0.001). Multivariate Cox regression analysis and prediction error analyses confirmed that EASIX remained a significant predictor of survival after adjustment for age, sex, cytogenetic abnormalities and bone marrow blasts in lower-risk patients. The model of the training cohort could be validated. Serum levels of Angiopioetin-2 correlated significantly with EASIX. We introduce EASIX as an easily accessible and independent predictor for survival in patients with lower-risk MDS.

## Introduction

Myelodysplastic syndromes (MDS) are a heterogeneous group of malignant clonal hematopoietic stem cell disorders characterized by morphological dysplasia, ineffective hematopoiesis and an increased risk of transformation into secondary acute myeloid leukemia (AML)^1,2^. Older patients are especially affected by this disease^3^.

Median survival time ranges from a few months to years, therefore estimation of prognosis is important at primary diagnosis^1,2^. The International Prognostic Scoring Systems (IPSS) and Revised International Prognostic Scoring System (IPSS-R) enable prediction of survival and risk of AML development by taking into account bone marrow cytogenetics, bone marrow blast count and cytopenias^1^. Five prognostic risk categories can be defined, with a median survival of 8.8 years in the very low-risk group and 0.8 years in the very high-risk group^1^.

Patients with low and intermediate-1 according to IPSS or very low and low-risk disease according to IPSS-R are considered lower-risk MDS^1^ and are usually not subjected to systemic therapy. Symptom-orientated supportive treatment (blood transfusions, erythrocyte stimulating agents, iron chelators) and regular monitoring are appropriate strategies for lower-risk patients^4^. However, life expectancy among patients with lower-risk MDS is significantly shorter compared to healthy individuals. Beyond progression into AML and complications resulting from cytopenias, cardiovascular complications represent a major risk of death in lower-risk MDS patients. For patients having been diagnosed with lower-risk MDS for more than 5 years, the cumulative mortality risk of cardiovascular disease even equals the mortality risk attributed to MDS^5^. One possible explanation may be heart disease due to iron overload. Another explanation might be the presence of cytokines mediating both stress hematopoiesis and systemic endothelial distress. The presence of monoclonal hematopoiesis has been associated to cardiovascular diseases and myocardial infarction^5–8^. Recently, clonal hematopoiesis of indeterminate potential (CHIP) has been identified as risk factor for impaired survival in patients with chronic ischemic heart failure^9^. Furthermore, the link between mutations in CHIP driver genes, inflammation, and development of artherosclerosis has been established in animal models^8–12^.

We recently established EASIX (Endothelial Activation and Stress Index, [creatinine × LDH]/platelets) for prediction of endothelial complications and mortality after allogeneic stem cell transplantation and after acute Graft-versus-Host Disease (GvHD). EASIX is an endothelial dysfunction related biomarker, that can be easily obtained with routine laboratory markers^13,14^.

In this study, we analyzed whether EASIX can predict mortality in higher-risk and in lower-risk MDS patients. Since allogeneic transplantation (alloSCT) can alter the natural history of MDS and may be an independent contributor to endothelial complications, this analysis was restricted to patients who did not undergo alloSCT during the course of their disease.

## Patients and methods

### Patients

Two independent cohorts were used for training and validation of EASIX as predictor of survival in patients with MDS. Patients from the training cohort (*n* = 193) were diagnosed and followed-up at the University Hospital Heidelberg, patients from the validation cohort (*n* = 338) were managed at the University Hospital Düsseldorf. Included were all patients with MDS who were treated in the two institutions between 1997 and 2017, had EASIX data available at the time of diagnosis, and for any reason never underwent alloSCT during the course of the disease. Risk stratification was performed according to IPSS-R and if not available to IPSS^1^. For the purposes of this study, patients with low and intermediate-1 according to IPSS and very low and low-risk according to IPSS-R^1^, respectively, were considered to have lower-risk MDS, while patients with intermediate-2 and high-risk according to IPSS and intermediate, high and very-high according to IPSS-R^1^, respectively, were considered to have higher-risk MDS^1^. Written informed consent to sample and data collection according to the declaration of Helsinki was obtained in all eligible patients. The responsible institutional review board approved sample and data collection as well as analysis.

### Assessment of serological markers of endothelial activation

Plasma levels of endothelial markers were measured in 74 patients of the training cohort at initial presentation and stored at −80 °C. HMGB1 was measured by using enzyme-linked immunosorbent assays (ELISA) (anti-HMGB1 antibody: Abnova, Taiwan; Horseradish Peroxi dase: Jackson ImmunoResearch Europe Ltd., UK; Standard ELISA Kit: Shino-Test Corporation, Japan). Angiopioetin-2 (ANG2), suppressor of tumorigenicity (ST)-2, soluble thrombomodulin (sCD141, BDCA3), interleukin (IL)-18, IL37, IL-1β and were measured by using commercial ELlSA (DuoSet®ELISA, Biotechne R&D, USA) according to the manufacturer’s descriptions.

### Statistical analysis

As described previously, EASIX at the time of primary diagnosis was calculated by the formula: LDH (U/l) x Creatinine (mg/dl) / Platelets (nl). The log_2_ transformed index was used for the statistical analysis. Survival was estimated using the Kaplan-Meier method. Overall survival (OS) was defined by time from primary diagnosis to death. Patients alive at the time of last follow-up were censored. For building prediction models, missing values in the data were imputed using the mice R package^15^. Univariate and multivariate Cox regression models were used to predict overall survival based on the EASIX score. The prediction performance was assessed by resampling techniques (bootstrap) and on the external validation cohort. We report Brier scores as a measure of time dependent prediction error. The discrimination, i.e. the ability of the model to distinguish patients with poor and good prognosis, is further assessed as described by Royston et al.^16^. Good discrimination is indicated if the regression coefficient of the linear predictor (prognostic index) as only regressor in the validation data is close to 1. Further, model misspecification is assessed by building a new model on the validation data with the prognostic index estimated in the training data added as offset. All regression coefficients should then be close to zero. P-values < 0.05 were considered significant without corrections for multiple testing. Analyses were performed using R statistical software [R Core Team (2019). R: A language and environment for statistical computing. R Foundation for Statistical Computing, Vienna, Austria. URL https://www.R-project.org/.].

Correlation of EASIX with endothelial serum markers was performed according to Spearman. Correlation of EASIX quartiles and ANG2 was assessed using the Jonckhere-Terpstra method.

## Results

### Patient characteristics

Patient characteristics by cohort and risk group are given in Table 1. In both cohorts, the majority of patients had lower-risk disease, while 32% (training cohort) and 23% (validation cohort) had higher-risk MDS. Comparison of baseline characteristics between lower-risk patients from both cohorts revealed significant differences for median platelet counts (training 164/nl vs validation 142/nl, *p* = 0.002) and EASIX (training 1.2 vs validation 1.6, *p* = 0.012). In higher-risk patient, significant differences between both cohorts were found for LDH (training 287 vs 224, *p* = 0.004). No significant differences between both cohorts according to risk status were assessed for all other baseline clinical parameters.

**Table 1:** Patient characteristics

	Cohort							
	Training Heidelberg				Validation Dusseldorf			
Total	193				333			
Risk group^a^	Lower		Higher		Lower		Higher	
*n*/ %	132	68%	60	32%	255	77%	78	23%
Sex male	82	62%	34	57%	150	59%	50	64%
Blasts ≥ 10%	8	6%	46	77%	3	1%	54	69%
Transfusions	29	22%	25	42%	143	56%	58	74%
	Median	Range	Median	Range	Median	Range	Median	Range
Age	70	33–84	73	47–86	70	20–91	71	47–84
LDH	222	100–670	287	117–992	214	105–881	224	155–542
Platelets	164	10–825	74	9–519	142	6–648	80	16–714
Creatinine	0.9	0.5–8.2	0.9	0.5–2.2	0.9	0.5–4.3	1.0	0.6–1.9
Hemoglobin	10.0	5.0–16.4	9.1	5.9–12.3	10.2	6.0–15.4	9.5	3.7–13.4
ANC	2,2	0.2–12.3	1.0	0–10.5	2.1	0.1–33.4	1.1	0.1–8.1
EASIX	1.2	0.1–65.0	3.3	0.3–47.7	1.6	0.2–130.2	3.0	0.2–188.6

*ANC* acute neutrophil count, *LDH* lactate dehydrogenase

^a^Risk stratification was performed according to IPSS-R and if not available to IPSS (lower-risk = low and intermediate-1 risk according to IPSS and very low and low-risk according to IPSS-R; higher-risk = intermediate-2 and high-risk according to IPSS and Intermediate, high and very-high according to IPSS-R)

### Univariate survival analysis

**Fig. 1:**
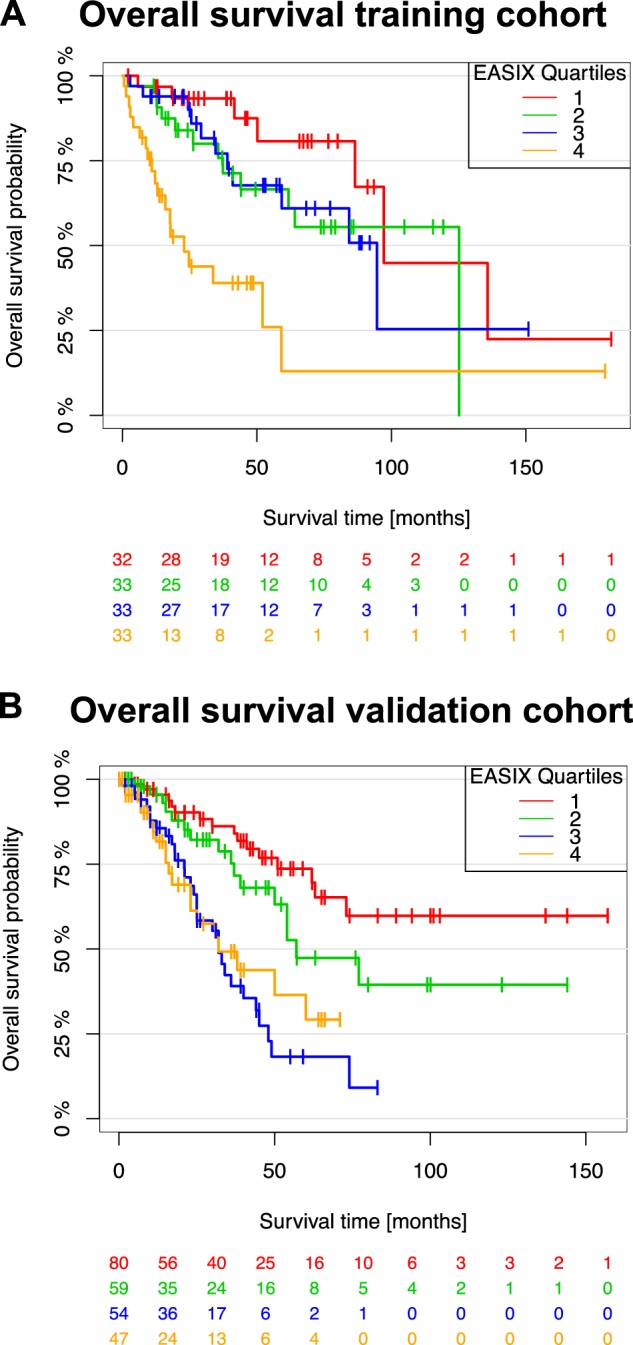
High EASIX associates with low overall survival in lower risk MDS patients. A Overall survival for patients with lower-risk MDS in the training cohort (**a**) and validation cohort (**b**) according to EASIX quartiles. Higher EASIX quartiles correlate with shorter survival in the training and validation cohort

One patient in the training cohort and 15 patients in the validation cohort were lost to follow-up. Median follow-up of lower-risk patients in the training cohort was 49 months and 40 months in the validation cohort. During follow-up, 49 events occurred in the training cohort and 87 events in the validation cohort, resulting in a median OS of 87 months and 54 months, respectively. In the higher-risk cohort, 54 events occurred in the training cohort and 52 events in the validation cohort, resulting in a median OS of 11 months and 12 months, respectively. Causes of death were known for 24 events in the lower risk training cohort (AML *n* = 7, other *n* = 17, unknown *n* = 15), whereas AML development was reported in 49 patients in the lower risk validation cohort. Univariate analyses revealed that a higher EASIX was associated with shorter OS in patients with lower-risk MDS from both cohorts (training: hazard ratio (HR) per log2 increase 1.46; 95% confidence interval (CI) 1.24–1.71; *p*-value < 0.001/validation: HR 1.31 [1.17–1.48]; *p*-value < 0.001). No effect of EASIX on survival was found in higher-risk patients from both cohorts (training: HR 1.11 [0.93–1.33]; *p*-value = 0.241/validation: HR 1.05 [0.91–1.21]; *p*-value = 0.493). We visualized this continuous effect of EASIX in lower-risk disease by grouping patients into quartiles according to EASIX (Fig. 1a, b). In both cohorts, patients in the highest quartile had a shorter survival compared to patients in lower quartiles. Confining our analysis to lower-risk patients who did not develop AML within the observation period, EASIX similarly predicted OS in both cohorts (training: *n* = 78, no. of events *n* = 17, HR per log2 increase 1.43 [1.05–1.94]; *p* = 0.02; validation: *n* = 267, no. of events *n* = 93, HR per log2 increase 1.33 [1.21–1.47]; *p* < 0.001).

Brier scores as a measure of time-dependent prediction errors were assessed in the training cohort. We found that the prediction error of the model with estimated effect of EASIX in the training cohort is preferable over the naive Kaplan–Maier estimate (Supplementary Fig. 1A). Model validation by transferring the univariable EASIX effect from the training to the validation model revealed that the prediction error of the model with newly estimated effect of EASIX and the model for which the effect of EASIX was transferred from the training set are preferable over the naive KM estimate. EASIX is, therefore, a validated predictor of mortality in lower-risk patients. We integrated EASIX-MDS into our online EASIX calculator (http://biostatistics.dkfz.de/EASIX/).

### Multivariate survival analysis

Multivariate Cox regression analyses confirmed that higher EASIX was correlated to shorter survival in the whole cohort with age, gender, transfusion dependence, and IPSS/R score as confounding variables (Supplementary Table 1). After subgrouping patients according to IPSS/R risk, EASIX was specifically associated with mortality in lower-risk patients from both cohorts, regardless of age, gender, cytogenetic abnormalities, and bone marrow blast counts. Again, no significant effects of EASIX on survival in higher-risk patients were found in patients from both cohorts. Table 2 summarizes results from multivariate analyses for both cohorts. Beyond EASIX, age, the presence of cytogenetic abnormalities and bone marrow blast counts above 10% were independent predictors of shorter survival in lower-risk patients.

**Table 2:** Multivariate analysis

	Training				Validation			
	Heidelberg				Dusseldorf			
*Lower-risk*								
	HR	Lower 95%CI	Upper 95%CI	*p*	HR	Lower 95%CI	Upper 95%CI	*p*
Sex (female)	0.67	0.33	1.37	0.27	0.96	0.63	1.48	0.87
Age	1.12	1.06	1.17	<*0.001*	1.05	1.02	1.07	<*0.001*
Cytogenetics	0.81	0.40	1.65	0.57	1.38	0.88	2.15	0.16
Blasts (>10%)	9.90	3.65	26.82	<*0.001*	0.94	0.13	6.94	0.96
Log_2_(EASIX)	1.33	1.12	1.59	*0.001*	1.41	1.24	1.59	<*0.001*
*Higher-risk*								
	HR	Lower 95%CI	Upper 95%CI	*p*	HR	Lower 95%CI	Upper 95%CI	*p*
Sex (female)	1.47	0.71	3.01	0.30	0.99	0.54	1.84	0.98
Age	1.01	0.96	1.05	0.71	1.02	0.99	1.05	0.28
Cytogenetics	0.77	0.37	1.62	0.49	1.55	0.80	3.00	0.20
Blasts (>10%)	1.36	0.65	2.85	0.42	1.49	0.77	2.87	0.24
Log_2_(EASIX)	1.15	1.94	1.40	0.18	0.99	0.85	1.16	0.94

Italic values indicates risk stratification was performed according to IPSS-R and if not available to IPSS (lower-risk = low and intermediate-1 risk according to IPSS and very low and low-risk according to IPSS-R; higher-risk = intermediate-2 and high-risk according to IPSS and Intermediate, high and very-high according to IPSS-R)

There was a strong model misspecification for the multivariable effects of all confounders in the lower-risk cohorts (Supplementary Table 2). In contrast, the effect of EASIX did not differ in the multivariable models of training and validation lower-risk cohort (*p* = 0.403). We, therefore, assessed the Brier score as a measure of time-dependent prediction errors for the validation cohort by newly estimating all confounders and transferring only the EASIX effects from the training cohort. Again, the prediction error of the multivariable model including the estimated effect of EASIX was shown to be preferable over the naive Kaplan–Maier estimate in lower-risk MDS (Supplementary Fig. 2).

### Correlation of cardiovascular comorbidities with iron overload and EASIX

Iron overload is another possible reason for cardiovascular complications in patients with MDS. Therefore, we analyzed cardiovascular comorbidities in the training cohort and retrieved ferritin serum levels from routine lab analyses. We were able to get a detailed history of cardiovascular comorbidities in 157 patients of the training cohort. A positive history was found in 88 patients (56%). While no significant differences in baseline serum ferritin values were observed between both groups (485 ng/ml versus 405 ng/ml; *p* = 0.81), patients with a positive history had higher baseline EASIX values than patients without any history of cardiovascular disease (2.0 versus 1.3; *p* < 0.01). Suppl. Table 3 reveals that EASIX remained significantly associated with mortality in lower-risk MDS if cardiovascular comorbidities and Ferritin were included in the multivariable analysis.

### Correlation of serological endothelial stress markers with EASIX

**Fig. 2:**
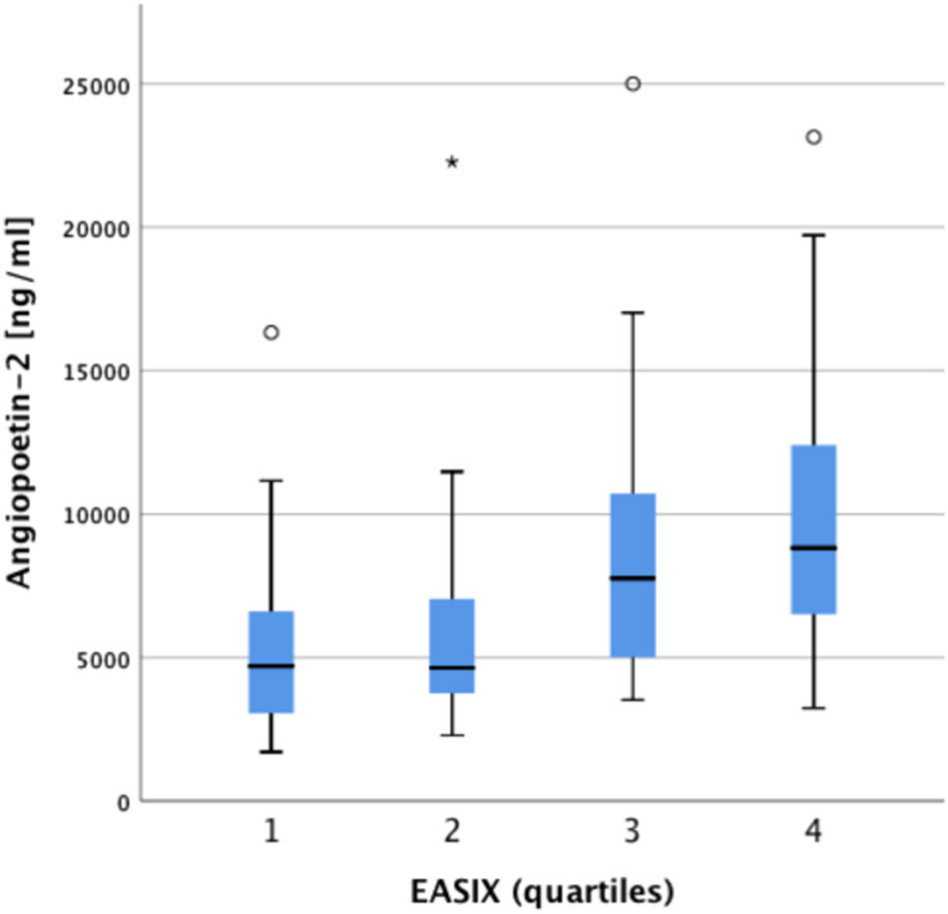
Correlation between serological endothelial stress markers and EASIX. Higher serum levels of Angiopoietin-2 correlate significantly with higher EASIX

Correlations between EASIX and endothelial serum markers at initial diagnosis are shown in suppl. Table 4. Weak correlations were observed between EASIX, sCD141 (soluble thrombomodulin) and IL-18, whereas ANG2 was strongly correlated (Spearman’s rank correlation = 0.456, 95%CI 0.264–0.616, *p*-value < 0.001, Supplementary Table 4). The relation of EASIX (quartiles) and ANG2 is visualized in Fig. 2. Due to the lack of correlation between EASIX and S100A9 or IL-1β we performed a more extensive pathway analysis including serum HMGB1 and IL37. A strong intra-pathway correlation of S100A9 with IL1β, IL18, IL37, and HMGB1 was observed, arguing for the validity of the serum analyses (Supplementary Table 5).

## Discussion

This retrospective cohort analysis demonstrates and validates that EASIX predicts overall survival in transplant-ineligible patients with lower-risk MDS. EASIX is calculated by assessment of three parameters (LDH, platelets, creatinine) that are usually measured in every routine workup. The strong correlation of EASIX and ANG2 underlines the endothelial nature of this marker. Therefore, EASIX may represent a broadly available tool to estimate overall survival in lower-risk MDS, independent of gender, age, cytogenetic abnormalities and bone marrow blast counts.

In the past, mortality of lower-risk MDS was mainly attributed to complications from cytopenias such as infections, bleedings, and transfusion-related disorders, as well as secondary transformation into AML. However, in recent years awareness is rising that lower-risk MDS patients succumb to cardiovascular complications more frequently than to secondary AML or disease related problems^6,7^. In line with this it is noteworthy that patients with clonal hematopoiesis of indeterminate potential (CHIP) are prone to cardiovascular diseases and deaths from myocardial infarction or heart failure^7–9^. With increasing time interval since MDS diagnosis the risk of cardiovascular events rises and equals the risk of transformation into AML after 5 years^5^.

A relationship between endothelial dysfunction and clonal hematopoiesis has been observed in a murine model of atherosclerosis^10^. The proposed mechanism for the link between clonal hematopoiesis and atherosclerosis involves increased NLRP3 inflammasome-mediated IL1β secretion in TET2-deficient macrophages in mice^10^. This is in line with findings showing that S100A9-mediated NLPR3 inflammasome activation is associated with pyroptosis in bone marrow of MDS patients, suggesting that this mechanism may account for a number of clinical features of MDS^17–19^. In order to delineate the pathogenesis of MDS-related endothelial dysfunction suggested by the prognostic impact of EASIX, we measured serum levels of S100A9, IL1β and IL18 (both activated by caspase 1 from pro-cytokines). We observed a weak correlation of EASIX with IL18 as previously reported for EASIX measured before conditioning for allogeneic stem cell transplantation^20^. In contrast, serum S100A9 and IL-1β did not correlate with EASIX, although S100A9 strongly correlated with the related cytokines IL1β, IL18, IL37 and HMGB1^21–23^. It is possible that NLRP3-mediated alterations of hematopoiesis and atherosclerosis are regulated in a local rather than a systemic way. However, systemic activity of the S100A9-NLPR3 pathway was demonstrated in this study, and it was unrelated to EASIX and outcome. In contrast, serum ANG2 as a systemic endothelial cell derived stress biomarker^24^ strongly correlated with EASIX.

Our study has limitations that need to be considered. First, due to the retrospective character of the analysis, only limited information was available on the exact causes of death in both cohorts. Therefore, it remains unclear if the observed higher mortality in patients with a higher EASIX was indeed caused by cardiovascular complications. Furthermore, the three parameters needed for calculation of EASIX are not specific for endothelial cell dysfunction on their own. They are affected by a multitude of physiological and pathological processes like cell turnover (LDH), insufficient or replaced hematopoiesis (platelets) as well as body mass and kidney function (creatinine). Another limitation that needs to be mentioned is the incomplete information on supportive care, e.g. application of hematopoietic growth factors and iron chelators.

Nevertheless, cardiovascular diseases are the second most common cause of death in lower-risk patients after MDS-related complications^5^ and EASIX predicted survival in two independent cohorts regardless of disease-specific factors associated with risk of progression into AML (cytogenetic abnormalities and blast count). Furthermore, subgroup analyses confined to patients who did not develop secondary AML within the observation period revealed a persistent predictive effect of EASIX. We also found no correlation between EASIX and mortality in higher-risk patients, where progression into AML is the major contributor to mortality. Lastly, analysis of cardiovascular comorbidities in the training cohort revealed significantly elevated EASIX values in patients with a history of cardiovascular disease. We did not find evidence for iron overload as possible confounder for cardiac dysfunction in our patients. This supports our hypothesis that EASIX may be linked to cardiovascular comorbidities and mortality.

Despite recent developments of targeted therapies in patients with lower-risk MDS^25^, watchful waiting and supportive treatment are appropriate strategies for lower-risk MDS patients^26^. In this study, we establish EASIX as a broadly available marker predicting shorter survival in lower-risk MDS. There is some likelihood that an increased risk of cardiovascular deaths contributes to the excess mortality indicated by higher EASIX. Therefore, EASIX identifies patients who might particularly benefit from closer monitoring and adjustment of cardiovascular risk factors, e.g. blood pressure, elevated serum lipids, body weight and blood sugar.

In conclusion, EASIX could be validated as an independent predictor of survival in patients suffering from lower-risk MDS.

## Supplementary information

Supplemental material
