# Supplementary material for: EASIX for prediction of survival in lower-risk myelodysplastic syndromes
Source: Blood Cancer J. 2019 Nov 11;9(11):85. doi: 10.1038/s41408-019-0247-z (PMC6848148; doi:10.1038/s41408-019-0247-z)
Supplement: Supplementary file 1 — Supplemental material [file 41408_2019_247_MOESM1_ESM.docx]

**Supplemental Table 1:** Multivariate analysis accounting additionally for transfusion-dependence and IPSS/-R.

| **Training cohort** | | | | | | |
| --- | --- | --- | --- | --- | --- | --- |
| **All patients** | | | | | | |
|  | **HR** | | **Lower 95%CI** | **Upper 95%CI** | | **p** |
| Log_2_(EASIX) | 1.16 | | 1.02 | 1.32 | | *0.023* |
| Age | 1.06 | | 1.03 | 1.09 | | *<0.001* |
| Transfusions | 1.26 | | 0.81 | 1.94 | | 0.303 |
| Gender (female) | 0.82 | | 0.52 | 1.29 | | 0.397 |
| IPSS-R very low=ref |  | |  |  | |  |
| IPSS-R low (2 points) | 2.04 | | 0.70 | 5.93 | | 0.191 |
| IPSS-R low (3 points) | 5.13 | | 1.74 | 15.15 | | *0.003* |
| IPSS-R intermediate | 9.46 | | 3.23 | 27.71 | | *<0.001* |
| IPSS-R high | 19.98 | | 6.04 | 66.09 | | *<0.001* |
| **Validation cohort** | | | | | | |
| **All patients** | | | | | | |
| Log_2_(EASIX) | 1.11 | 1.00 | | 1.22 | *0.044* | |
| Age | 1.03 | 1.01 | | 1.05 | *0.009* | |
| Transfusions | 1.62 | 1.08 | | 2.42 | *0.020* | |
| Gender (female) | 1.01 | 0.71 | | 1.44 | 0.960 | |
| IPSS low=ref |  |  | |  |  | |
| IPSS intermediate-1 | 1.69 | 1.06 | | 2.69 | *0.026* | |
| IPSS intermediate-2 | 4.21 | 2.52 | | 7.03 | *<0.001* | |
| IPSS high | 4.76 | 2.13 | | 10.68 | <0.001 | |

**Supplemental Table 2: Model misspecification (multivariable model)**

Results of multivariate Cox regression analysis to check model misspecification – complete model transferred to validation cohort. The effects of nearly all confounders differ significantly from what was observed in the training cohort with the exception of EASIX. CI = confidence interval

| **Parameter** | **Coefficient** | **Lower 95% CI** | **Upper 95% CI** | **p-value** |
| --- | --- | --- | --- | --- |
| Log_2_(EASIX) | 1.05 | 0.93 | 1.19 | 0.403 |
| **Blasts > 10%** | **0.10** | **0.01** | **0.70** | **0.021** |
| **Age** | **0.94** | **0.92** | **0.96** | **<0.001** |
| **Sex female** | **0.65** | **0.42** | **0.99** | **0.047** |
| **High risk cytogenetics** | **1.69** | **1.09** | **2.64** | **0.020** |

**Supplemental Table 3.** Multivariate analysis accounting also for cardiovascular disease (CVD) and serum ferritin. CHD = coronoary heart disease, PAD = Peripheral artery disease

| **Training cohort** | | | | | |
| --- | --- | --- | --- | --- | --- |
| **All patients** | | | | | |
|  | | **HR** | **Lower 95%CI** | **Upper 95%CI** | **p** |
| Log_2_(EASIX) | | 1.20 | 1.05 | 1.37 | *0.008* |
| Age | | 1.07 | 1.03 | 1.10 | *<0.001* |
| Transfusions | | 1.12 | 0.72 | 1.74 | 0.622 |
| Gender (female) | | 0.86 | 0.54 | 1.36 | 0.512 |
| IPSS-R very low=ref | |  |  |  |  |
| IPSS-R low (2 points) | | 1.99 | 0.67 | 5.89 | 0.214 |
| IPSS-R low (3 points) | | 5.29 | 1.77 | 15.79 | *0.003* |
| IPSS-R intermediate | | 10.27 | 3.47 | 30.37 | *<0.001* |
| IPSS-R high | | 19.12 | 5.73 | 63.88 | *<0.001* |
| CVD (CHD, PAD) | | 0.81 | 0.49 | 1.36 | 0.432 |
| CVD (other) | | 0.57 | 0.34 | 0.94 | *0.029* |
| Ferritin | | 1.00 | 1.00 | 1.00 | 0.064 |
| **Lower-risk MDS** | | | | | |
| Log_2_(EASIX) | | 1.32 | 1.10 | 1.59 | *0.003* |
| Age | | 1.11 | 1.06 | 1.17 | *<0.001* |
| Transfusions | | 1.09 | 0.52 | 2.28 | 0.824 |
| Gender (female) | | 0.57 | 0.27 | 1.17 | 0.127 |
| IPSS-R very low=ref | |  |  |  |  |
| IPSS-R low (2 points) | | 2.22 | 0.71 | 6.91 | 0.168 |
| IPSS-R low (3 points) | | 5.89 | 1.91 | 18.10 | *0.002* |
| IPSS-R intermediate | |  |  |  |  |
| IPSS-R high | |  |  |  |  |
| CVD (CHD, PAVK) | | 0.55 | 0.23 | 1.32 | 0.184 |
| CVD (other) | | 0.45 | 0.22 | 0.93 | *0.030* |
| Ferritin | | 1.00 | 1.00 | 1.00 | 0.618 |
| **Higher-risk MDS** | | | | | |
| Log_2_(EASIX) | 1.06 | | 0.87 | 1.31 | 0.558 |
| Age | 1.00 | | 0.95 | 1.05 | 0.977 |
| Transfusions | 0.78 | | 0.41 | 1.46 | 0.438 |
| Gender (female) | 1.64 | | 0.82 | 3.28 | 0.165 |
| IPSS-R high=ref | |  |  |  |  |
| IPSS-R low (2 points) | |  |  |  |  |
| IPSS-R low (3 points) | |  |  |  |  |
| IPSS-R intermediate | | 0.41 | 0.20 | 0.86 | *0.018* |
| CVD (CHD, PAVK) | 1.27 | | 0.62 | 2.62 | 0.511 |
| CVD (other) | 1.13 | | 0.50 | 2.52 | 0.771 |
| Ferritin | 1.00 | | 1.00 | 1.00 | *<0.002* |

**Supplemental Figure 1: Univariable model – individual prediction errors in both cohorts.** Brier scores were calculated as a measure of time-dependent prediction errors for both cohorts independently. Prediction error of the model with estimated effect of EASIX is preferable over the naive Kaplan-Maier estimate (black lines) in both cohorts. **(A) Training cohort:** orange line = Prediction error with effect of EASIX **(B) Validation cohort:** red line = Prediction error with effect of EASIX newly estimated in validation cohort. Green line = Prediction error with effect of EASIX transferred from the training cohort. No data splitting, performance with independent training and validation cohorts.

**
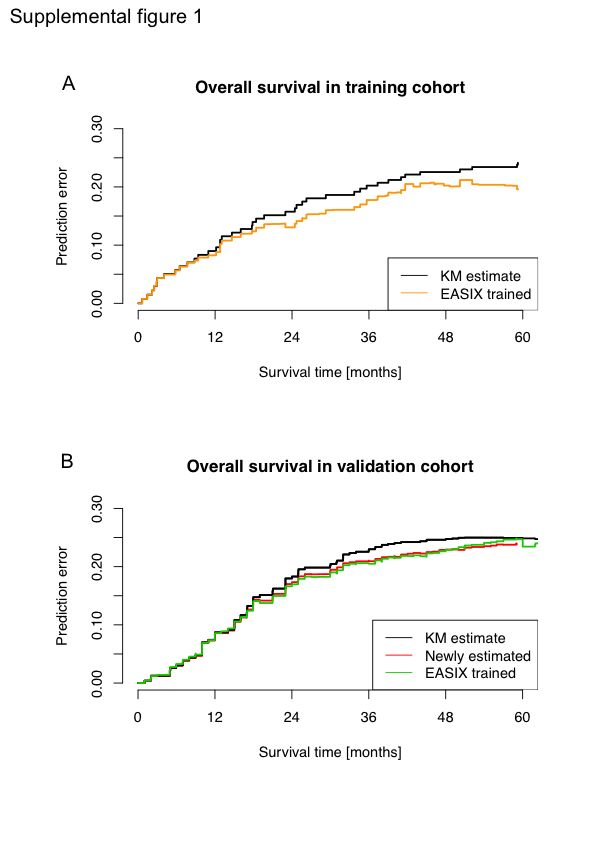
**

**Supplemental Figure 2: Multivariable model – prediction errors in validation cohort.** Prediction error of the model with estimated effect of only EASIX is also preferable over the naive Kaplan-Maier estimate (black line) in the multivariate model. Blue line = Prediction error estimated in the validation cohort with effect of all variables transferred from the training cohort. Green line = Prediction error estimated only with effect of EASIX transferred from the training cohort. No data splitting, performance with independent training and validation cohorts.

**
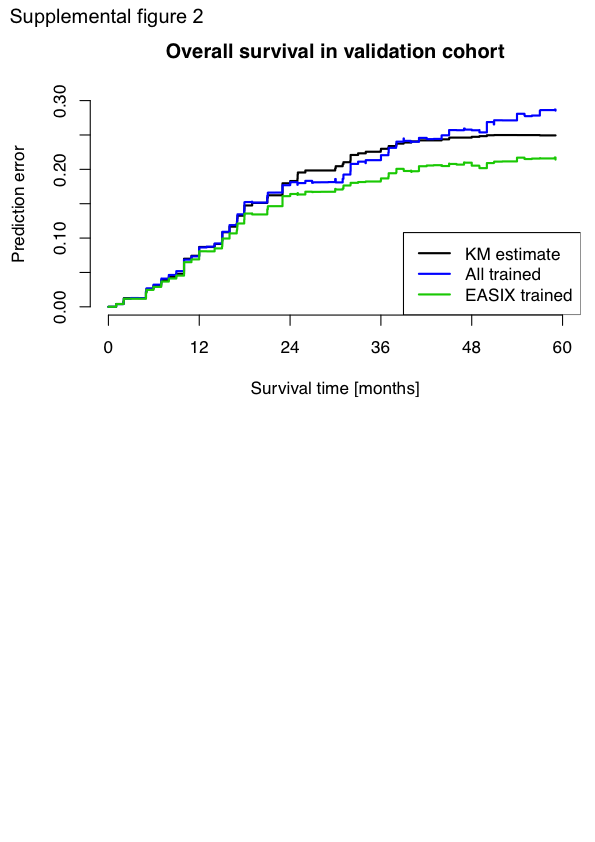
Supplemental Table 4**

**Correlation between EASIX and endothelial serum markers at initial diagnosis**

ST-2 = IL33-receptor; sCD141 = soluble thrombomodulin; ANG2 = Angiopoietin2; IL = Interleukine; S100A9 = S100 calcium-binding protein A9; HMGB1 = High-Mobility-Group-Protein B1. Correl.coeff. = correlation coefficient.

| **EASIX vs.** | **ST-2** | **sCD141** | **ANG2** | **IL-18** | **S100A9** | **IL-1b** | **IL-37** | **HMGB1** |
| --- | --- | --- | --- | --- | --- | --- | --- | --- |
| **correl.coeff.** | 0.063 | 0.205 | **0.456** | 0.237 | -0.394 | -0.027 | -0.141 | -0.075 |
| **p (2-sided)** | 0.530 | 0.072 | **<0.001** | 0.039 | 0.391 | 0.820 | 0.393 | 0.650 |
| **n** | 100 | 78 | 74 | 76 | 86 | 73 | 39 | 39 |

**Supplemental Table 5**

**intra-pathway correlation of S100A9 with IL18, IL1β, IL37 and HMGB1**

S100A9 = S100 calcium-binding protein A9; IL = Interleukine; HMGB1 = High-Mobility-Group-Protein B1

| **S100A9 vs.** | **IL-18** | **IL-1 β** | **IL-37** | **HMGB1** |
| --- | --- | --- | --- | --- |
| **correl.coeff.** | **0.351** | **0.276** | **0.769** | **0.620** |
| **p (2-sided)** | **0.002** | **0.018** | **<0.001** | **<0.001** |
| **n** | **75** | **73** | **39** | **39** |
